# Clinical applications of machine learning for infection assessment in diabetic foot ulcers

**DOI:** 10.3389/fphys.2026.1776883

**Published:** 2026-03-16

**Authors:** Khrystyna Lysnychka, Larysa Rostoka, Yana Burmistrova, Iryna Halabitska, Pavlo Petakh, Oleksandr Kamyshnyi

**Affiliations:** 1 Department of Biochemistry and Pharmacology, Uzhhorod National University, Uzhhorod, Ukraine; 2 Department of Therapy and Family Medicine, I. Horbachevsky Ternopil National Medical University, Ternopil, Ukraine; 3 Department of Microbiology, Virology, and Immunology, I. Horbachevsky Ternopil National Medical University, Ternopil, Ukraine

**Keywords:** artificial intelligence, clinical decision support, diabetic foot infection, diabetic foot ulcer, digital wound imaging, machine learning, medical image analysis, telemedicine

## Abstract

Diabetic foot ulcers (DFUs) represent one of the most severe complications of diabetes mellitus and are frequently complicated by infection, which significantly increases the risk of hospitalization, lower-limb amputation, and mortality. Early and accurate detection of infection in DFUs is therefore critical; however, clinical assessment remains challenging and is largely based on subjective visual evaluation. Inter-observer variability, atypical inflammatory responses in patients with diabetes, and inconsistent wound documentation contribute to delayed or inaccurate diagnoses. In recent years, digital sound imaging combined with machine learning (ML) techniques has emerged as a promising adjunct to traditional clinical assessment. This review summarizes and critically evaluates recent advances in the application of ML for infection assessment in DFUs. We review image-based ML approaches designed to detect infection-related visual features, including erythema, purulent exudate, necrosis, and tissue discoloration, as well as models developed for ulcer classification, tissue segmentation, and longitudinal wound monitoring. In addition, we discuss the clinical utility of ML-assisted tools in telemedicine, remote monitoring, and decision support, particularly in community and resource-limited settings. Current limitations, including image variability, dataset bias, lack of standardized imaging protocols, and limited clinical validation, are also addressed. Overall, ML-based systems have demonstrated encouraging performance in identifying infection-associated patterns in DFU images and may help reduce diagnostic variability and support earlier clinical intervention. Nevertheless, further large-scale prospective studies, regulatory validation, and integration into clinical workflows are required before widespread adoption. Machine learning should be viewed as a supportive tool that complements, rather than replaces, clinical expertise in the management of diabetic foot infections.

## 1 Introduction

Diabetic foot ulcers (DFUs) are a serious global health issue, affecting an estimated 18.6 million people worldwide each year (Armstrong et al., 2023). These wounds carry a high risk of infection and amputation: up to one-third of people with diabetes may develop a DFU in their lifetime, and DFUs precede about 80% of diabetes-related lower limb amputations (Luu et al., 2025). More than half of DFUs become infected over their course, and approximately 20% of those with moderate-to-severe infection ultimately require an amputation (Almufadi and Alhasson, 2024). Infections in DFUs greatly increase patient morbidity and mortality. For instance, one analysis noted 5-year mortality rates exceeding 50% following a major diabetes-related amputation–a prognosis on par with or worse than many cancers (Armstrong et al., 2020). Even in less severe cases, diabetic foot infections (DFIs) are associated with frequent hospitalizations, prolonged antibiotic therapy, and significant healthcare costs (Ramirez-Acuña et al., 2019; Sen and Demirdal, 2020). DFIs thus represent a major complication of diabetes, contributing to poor outcomes and straining healthcare systems globally (Guan et al., 2024; Edmonds et al., 2021).

Despite the clinical importance of infection in DFUs, assessing and diagnosing infection is challenging in practice. Clinicians typically rely on visual examination of the wound for signs of inflammation such as redness, warmth, swelling, pain, or purulent discharge (Lipsky et al., 2004). Guidelines (e.g., IWGDF/IDSA criteria) require at least two of these signs to define an active infection (Schaper et al., 2024). However, these traditional indicators can be subtle or absent in patients with diabetic neuropathy or ischemia, who may not mount normal inflammatory responses (Craus et al., 2023). As a result, early infection can be easily missed by visual judgment alone. Inter-observer variability further complicates clinical assessment–studies have shown that even experienced providers often disagree on the presence or severity of infection when examining the same wound, whether in person or via photographs (van Netten et al., 2017). For example, plain X-rays for suspected osteomyelitis in DFU have low sensitivity and their interpretation is highly operator-dependent, with poor interrater agreement without additional context (Álvaro-Afonso et al., 2013). Such subjective variability in diagnosing DFU infection can delay necessary treatment in some cases and lead to overuse of antibiotics or invasive procedures in others (Llewellyn et al., 2020). In summary, the visual assessment of infection is limited by inconsistency and the often atypical presentation of DFIs in neuropathic patients. There is a clear clinical need for more objective and reliable methods to detect and quantify infection in DFUs.

Digital photography and telemedicine have emerged as useful tools to address these gaps in DFU care (Debnath et al., 2025). High-resolution wound images allow documentation of ulcer appearance over time and enable remote expert consultation. In fact, telemedicine-based DFU monitoring during the COVID-19 pandemic showed outcomes comparable to traditional care while improving access (Méndez et al., 2025). Digital imaging can facilitate measurements of wound size and characteristics; for example, specialized wound image analysis systems achieve high reliability in measuring ulcer area and identifying surface changes (Bowling et al., 2011; Hazenberg et al., 2010; Foltynski et al., 2011). However, beyond gross measurements, nuanced signs of infection (e.g., subtle color changes or exudate) are difficult to evaluate consistently with the naked eye. Recent studies have reported that purely remote assessments of DFU photographs have low-to-moderate agreement for features like infection and exudate, underscoring the need for decision support (van Netten et al., 2017). This is where advanced algorithms and machine learning (ML) can play a transformative role. By extracting quantitative features from images and clinical data, ML can help standardize infection assessments that currently depend on subjective human judgement (Yogapriya et al., 2022; Shen et al., 2017).

Machine learning, particularly deep learning in image analysis, is rapidly being explored to improve DFU infection detection and related tasks. Infection Classification: Early proof-of-concept studies demonstrate that convolutional neural networks can classify DFU images as “infected” vs. “uninfected” with promising accuracy (Xu et al., 2022). For instance, the recent Diabetic Foot Ulcer Challenge 2021 provided a large dataset for classifying infection and ischemia from wound photos; top algorithms achieved a moderate F1-score ∼0.63, highlighting both the potential and current limitations of such models (Cassidy et al., 2022). Nonetheless, several groups report high technical performance in binary infection detection. In one study, AI models exceeded 90% accuracy for identifying DFUs with infection, comparable to expert clinicians. Xu et al. (2022) showed that deep learning methods can reliably detect infection and ischemia features from ulcer images, which is crucial for guiding treatment planning. These image-based classifiers can pick up subtle color and textural patterns (such as surrounding redness or purulence) that may elude the human eye, potentially alerting providers to infection earlier than visual inspection alone. Wound Segmentation: In parallel, ML algorithms have been developed to segment wound regions and tissue types on DFU images, automatically measuring wound area, depth, and detecting necrotic vs. granulation tissue (Tulloch et al., 2020). Such objective wound assessments can support infection evaluation (e.g., identifying necrosis or increasing wound size that might indicate uncontrolled infection) and allow tracking of healing progress over time. Risk Prediction and Prognosis: Beyond image analysis, machine-learning models using clinical features are being applied to predict important outcomes in DFU patients. Researchers have built prediction tools for amputation risk and healing failure–for example, models using patient and wound characteristics can forecast the likelihood of major amputation or sepsis in an infected DFU. Other studies have used routine biomarkers and clinical data to distinguish diabetic foot osteomyelitis from soft-tissue infection, providing decision support for clinicians on the need for bone biopsy or surgery (Yasin et al., 2025). Notably, recent work by Spinazzola et al. (2025) employed a deep recurrent neural network on serial wound assessments to predict healing trajectories in chronic DFUs, achieving about 80% accuracy in forecasting whether a wound will heal or deteriorate. Such prognostic ML models can help clinicians identify at-risk wounds (e.g., infections unlikely to respond to standard care) and personalize interventions early.

The integration of these advanced digital tools holds great promise for improving DFU outcomes. By enhancing early infection detection, reducing inter-observer variability, and providing data-driven prognostic insights, machine learning can augment clinical decision-making in diabetic foot care (Basiri, 2025). In effect, ML-driven analysis of wound photos and patient data can serve as a “second set of eyes” to support clinicians, standardizing infection assessments that currently vary between observers. It also enables remote monitoring: a smartphone image analyzed by an AI algorithm could flag infection changes between clinic visits, prompting timely interventions (Debnath et al., 2025). While these technologies are still in early stages, the convergence of digital photography and AI is poised to significantly advance the accuracy and consistency of DFU infection evaluation (Álvaro-Afonso et al., 2025).

The aim of this mini-review is to summarize and critically evaluate recent applications of machine learning for infection assessment and related diagnostic tasks in diabetic foot ulcers. We discuss current evidence from 2019 to 2025 on ML-based DFU image classification (especially infection and severity detection), wound segmentation, and outcome prediction, highlighting the clinical relevance, performance, and limitations of these approaches. Ultimately, leveraging ML for DFU infection assessment may improve early diagnosis, guide targeted therapies, and reduce the heavy burden of diabetic foot infections on patients and healthcare systems.

## 2 Clinical criteria for infection in diabetic foot ulcers

International guidelines such as those from the IWGDF and IDSA define diabetic foot ulcer (DFU) infection as a clinical diagnosis based on the presence of local signs of inflammation, rather than microbiological results alone (Senneville et al., 2024; Lipsky et al., 2012). In practice, a DFU is considered infected if at least two of the following signs are present: erythema–redness of the skin around the wound; warmth–increased local skin temperature; edema (swelling) or induration (hardening of the tissue around the ulcer); pain or tenderness in the area; purulent discharge–pus exuding from the ulcer (van Netten et al., 2024).

The mere isolation of bacteria from a wound (colonization) is not sufficient to diagnose infection in the absence of these clinical signs (National Institute for Health and Care Excellence: Guidelines, 2023). Notably, many patients with diabetic neuropathy may not feel pain in the ulcerated foot, so infection can be present even without pain; nonetheless, if pain is reported (or if the patient has new onset of tenderness), it is a concerning sign of possible deep or advancing infection (Schaper et al., 2020).

Importantly, the absence of systemic symptoms (such as fever, chills, elevated white blood cell count, or other signs of systemic inflammation) does not exclude a localized foot infection. The IDSA/IWGDF criteria emphasize that a DFU can be mildly or moderately infected based solely on local signs, without any systemic illness or fever (Elawady, 2025). Systemic inflammatory response signs are only expected in the most severe cases that have progressed beyond the foot. In other words, a patient with a red, swollen, warm ulcer with purulent exudate–meeting the local criteria–has an infection even if they feel well and remain afebrile. This distinction is crucial because diabetic individuals, especially those with peripheral neuropathy or peripheral arterial disease, might not mount a strong systemic response even when a limb infection is present (Lipsky et al., 2012).

Clinical guidelines further stratify DFU infections by severity (mild, moderate, or severe) based on the extent of the local infection and the presence of any systemic findings. The categories can be defined as follows: Mild–Local infection involving only the skin and superficial subcutaneous tissue. The wound has ≥2 signs of local inflammation, but the erythema (cellulitis) around the ulcer is limited in extent (for example, ≤2 cm around the ulcer) and there are no systemic signs of illness. Moderate–Local infection with either greater extent or deeper involvement. This category includes cases where cellulitis or erythema extends >2 cm from the wound margin, or where the infection involves deeper structures (such as abscess, fascial spread, muscle, tendon, or bone), but still without systemic inflammatory response. Severe–An infection accompanied by signs of systemic toxicity or metabolic instability, indicating that the infection has spread systemically. The patient shows overt systemic signs of infection (e.g., fever, chills, tachycardia, hypotension, confusion or other sepsis-related manifestations). Even a patient who initially has a mild local infection can progress to a severe, limb-threatening infection if not promptly treated, underlining the need for early recognition and intervention (Wukich et al., 2013).

Visible vs. Non-Visible Signs (Implications for Remote Assessment): Most of the above infection criteria are visual or tactile cues intended for in-person clinical examination. Signs like erythema, swelling, and purulent discharge are directly visible and can often be appreciated in wound photographs, which is advantageous for telemedicine or automated image-based assessment (Yap et al., 2015). In contrast, warmth is detected by touch or thermometer and pain must be reported by the patient–neither is directly observable in a standard photograph (Søndergaard et al., 2023). This poses challenges for remote DFU evaluation: for example, an ulcer might appear only mildly red in a photo, while an examiner in person could feel significant warmth indicating infection (a sign that would be missed by looking at the image alone) (Søndergaard et al., 2023). Conversely, erythema in an image can be misinterpreted due to lighting or camera artifacts; poor lighting may obscure redness, whereas certain lighting conditions or filters might exaggerate it (Sait and Nagaraj, 2025). Skin tone is another critical factor–redness can be much less apparent on darker skin, potentially leading clinicians or algorithms to underestimate infection severity from photographs (Brown et al., 2025). Studies have noted that both human assessors and computer vision algorithms can struggle to detect inflammation on highly pigmented skin, necessitating adjusted approaches or calibration in imaging tools (Harrison, 2023).

These limitations are highly relevant for machine learning models aiming to assess DFU infection from images. Current algorithms can be trained to recognize visible infection signs (e.g., redness or wound exudate) in photographs, but they may miss infections that lack dramatic visual cues (Wang et al., 2015). For instance, a “mild” infection without obvious pus could be overlooked if the surrounding redness is subtle or if the patient’s skin is dark, leading to a false-negative assessment by an automated system (Yap et al., 2021). To improve remote detection, some research teams are exploring multi-modal strategies–for example, combining standard wound photographs with thermal imaging (to detect increased skin temperature) or with patient-reported symptoms (to include pain feedback) – in order to capture those non-visual indicators of infection (Dias de Oliveira et al., 2025). In clinical telemedicine practice, providers often compensate for the limitations of photos by asking patients or caregivers about warmth, pain, or malodor, and by using serial images to judge swelling or spreading erythema over time (Søndergaard et al., 2023). As advanced telehealth and AI tools evolve, incorporating such supplementary information and accounting for visual variability (due to lighting or skin pigmentation) will be essential to reliably identify infections from afar (Wang et al., 2015; Karthik et al., 2025).

## 3 Challenges in traditional diabetic foot ulcer infection assessment

Traditional assessment of infection in DFUs relies heavily on visual inspection and clinician judgment, which is inherently subjective. Studies indicate that evaluating wounds by appearance alone suffers from poor inter- and even intra-observer reliability (Karthik et al., 2025; Weigelt et al., 2022). In practice, different clinicians may disagree on infection severity or presence, as visual scoring is prone to individual interpretation. The PEDIS system, for example, still depends on subjective clinical judgment, leading to significant inter-observer variability and potentially delayed diagnoses (Chuan et al., 2015). Such variability underscores how non-standardized visual examinations can yield inconsistent assessments of infection.

When assessments are based on wound photographs (as in telemedicine or remote monitoring), additional challenges emerge. Recognizing infection from a photo alone is difficult even for experts. Extrinsic factors in imaging often confound the evaluation: differences in ambient lighting, camera quality, and photo angle can all alter the perceived wound characteristics. In the absence of a controlled imaging setup, the same wound might appear differently across images. Moreover, there is currently no universal standard protocol for wound photography. A lack of standardized image acquisition procedures means photos are taken at varying distances, lighting conditions, and with different devices, resulting in inconsistent image quality (Onuh et al., 2022). Indeed, a recent review noted that the absence of standardized imaging protocols–coupled with inconsistent equipment quality–hampers reliable wound assessment. These environmental and technical limitations reduce the objectivity of photo-based evaluations (Murphy et al., 2006).

Another significant challenge is the low reliability of photos in depicting specific signs of infection. Certain hallmark indicators of DFU infection are not easily or consistently captured in two-dimensional images (Sari et al., 2025). For example, erythema (redness of periwound skin) can be subtle and is often subject to misinterpretation on photos–one analysis found that erythema has very poor inter-observer agreement when assessed via photographs (Totty et al., 2018). Color accuracy may be distorted by lighting or camera sensors, making it hard to judge redness reliably. Similarly, purulent exudate (pus) or other wound secretions may not be clearly visible or distinguishable in images, especially if resolution is low or the photo is taken after cleaning the wound. Only a few studies have evaluated the detection of infection signs like exudate using images, and those report low diagnostic validity (van Netten et al., 2017; Rasmussen et al., 2015).

## 4 Machine learning approaches for infection assessment

Advances in machine learning (ML) are addressing the above challenges by providing more objective and consistent analyses of diabetic foot ulcers (DFUs). Modern ML techniques–especially deep learning with convolutional neural networks (CNNs) – can be trained on wound images to detect infection, often with high accuracy (Foltynski et al., 2025). These approaches fall into several categories, including direct image classification of infection, detailed tissue color analysis, and tools for tracking ulcer changes over time. By learning from large datasets, ML models aim to reduce observer variability and detect subtle patterns indicative of infection that clinicians might overlook (Table 1).

**TABLE 1:** Overview of AI-driven frameworks for diabetic foot ulcer diagnosis, perfusion monitoring, and severity scoring.

Study (Author, year)	Clinical aim	Dataset used	Infection definition	ML method (brief)	Performance metrics	Key clinical takeaway
Wang et al. (2024)	Develop a smart scoring system (ScoreDFUNet) for objective DFU severity evaluation	1,944 images (DFUC 2020, hospital data, Kaggle)	Categorization into soft tissue infections or deep abscesses/osteomyelitis based on Wagner’s system	U-Net (segmentation) + ResNet50 (classification) with transfer learning	95.34% detection accuracy	Surpasses junior dermatologists; provides consistent, objective scoring in ∼15 s
Rathore et al. (2025)	Enhance DFU identification with explainable AI (XAI) to build clinician trust	3,100 skin patches (normal vs. ulcer) after augmentation	Binary identification of ulcers (which are prone to infection) vs. healthy skin	Siamese Neural Network (SNN) + ResNet50 + XAI tools (SHAP, LIME, Grad-CAM)	98.76% accuracy; 98.5% F1-score	XAI heatmaps allow clinicians to visualize decision-making, reducing the “black box” nature of AI.
Gherardi et al. (2025)	Mobile blood perfusion monitoring using hyperspectral reconstruction from RGB images	∼6,000 images (DFUC 2021) and SPECTRALPACA videos	Sensitivity to infection measured through mean sigmoid oxygenation values	MobiPerf (MST++ architecture) for hyperspectral reconstruction (HSR)	Significant sensitivity to ischemia (p < 1e-5) and infection (p < 0.0001)	Enables monitoring using standard smartphones without specialized hardware or calibration targets
Xu et al. (2022)	Explicitly utilize class knowledge for infection/ischemia classification	DFU dataset from Lancashire Teaching Hospitals	Bacterial soft tissue or bone infection in the DFU.	Class Knowledge Banks (CKBs) with DeiT (Vision Transformer) encoder	78.00% accuracy (Infection); 90.90% accuracy (Ischemia)	CKBs effectively handle class imbalance by giving equal importance to different categories
Sendilraj et al. (2024)	Develop DFUCare platform for automated localization and macroscopic analysis	DFUC 2020 and 2021 datasets	Binary classification (infected vs. non-infected) based on expert clinical labels	YOLOv5s (localization) + InceptionResNetV2 (classification) + K-means (color)	79.76% accuracy (Infection); 94.81% (Ischemia)	Combines DL with textural/color analysis to provide a comprehensive remote monitoring tool
Liu et al. (2022)	Adapt EfficientNet for high-speed binary classification of infection and ischemia	DFUC 2021 dataset.	Presence of bacteria causing cell death in the wound	EfficientNet (B0-B7) utilizing compound scaling	98% accuracy (Infection); 99% accuracy (Ischemia)	Significantly faster inference (10%–50% of baseline time), making it ideal for real-time mobile assessment

### 4.1 Image-based classification models

One major approach uses CNN-based image classification to determine if a given ulcer image is infected or not (a binary classification). Early efforts in this area established the foundation by curating labeled datasets and applying both traditional and deep learning methods. Notably, Goyal et al. (2020) introduced one of the first public DFU image datasets with ground truth labels for infection and ischemia. Using handcrafted color features (a “superpixel color descriptor”) and an ensemble CNN, their system achieved about 73% accuracy in distinguishing infected vs. non-infected ulcers. This work highlighted that infection recognition from images is feasible, but initially less accurate than ischemia detection (ischemia classification reached 90% in the same study).

Subsequent studies rapidly improved performance by leveraging deep CNN architectures and larger training sets. Transfer learning with pre-trained networks (e.g., ResNet, EfficientNet) proved especially effective (Karthik et al., 2025). For instance, Liu et al. (2022) used EfficientNet models and extensive augmentation, achieving 98% accuracy for infection classification–a substantial jump from the ∼73% accuracy of prior models. The EfficientNet outperformed earlier ResNet and even the Goyal et al. ensemble, demonstrating the benefit of modern deep learning in this task. Likewise, researchers in 2022 proposed a specialized Diabetic Foot Infection Network (DFINET) with parallel convolutional layers, reporting ∼92% classification accuracy for detecting infection from images (Yogapriya et al., 2022). By 2024, hybrid models combining multiple CNNs have pushed performance further–one system (ScoreDFUNet) categorized images into normal, ulcer, infected, and gangrene regions with 95.3% accuracy, equaling expert-level assessments (Wang et al., 2024). Such results suggest that ML image classifiers can approach the consistency of experienced clinicians, while greatly accelerating the assessment process.

In developing these models, several open datasets and challenges have catalyzed progress. The MICCAI DFU Classification Challenge (DFUC 2020) provided over 4,000-foot images for training and testing algorithms. Participants designed novel CNN ensembles (e.g., DenseNet+MobileNet combinations) that achieved >95% accuracy in identifying DFU images (Debnath et al., 2025). While that particular challenge focused on ulcer vs. normal skin detection, its data have been repurposed for infection classification research. Consistent across studies is the use of performance metrics like sensitivity, specificity, and F1-score to evaluate models, given the high consequences of missed infections. For example, one deep ensemble achieved an F1 ∼0.85 for infection detection at the wound level. Overall, image-based ML classifiers show excellent potential as a diagnostic aid–flagging which ulcers appear infected on photos–with accuracies in recent studies generally in the 80%–95% range. Such tools can standardize infection assessment and alert providers to high-risk wounds, addressing the subjectivity of human evaluation.

### 4.2 Tissue and color analysis

Beyond coarse classification, ML is also used to analyze wound tissues and colors in images to infer infection status (Zhu et al., 2025). Infected DFUs often exhibit specific local changes–e.g., purulent exudate (yellowish slough or pus), necrotic tissue (black eschar), or surrounding erythema (redness) – which can be quantified by image analysis. Machine learning models have been developed to segment wound regions and classify the tissue composition by color/texture, providing a more granular assessment of infection-related features. For example, a model might identify what percentage of the wound area is covered by yellow fibrin (a possible sign of infection) versus healthy red granulation tissue (Cazzell et al., 2019). In a 2025 study, a transformer-based segmentation model could delineate not only the ulcer area but also identify regions of necrotic tissue, exposed bone, and surrounding erythema with high precision (Zhou et al., 2025). Such detailed tissue maps are clinically valuable: for instance, presence of necrosis signals the need for debridement, while the extent of erythema around a wound indicates the spread of infection/inflammation.

Color space analysis is a key component of these methods. Researchers have used transformed color spaces like CIELab or YCbCr to better separate relevant color features (e.g., differentiate red tones from lighting effects). The DFUCare platform (2024) exemplifies this approach: it combines wound localization with color segmentation to analyze macroscopic features of ulcers (Sendilraj et al., 2024). DFUCare applies unsupervised k-means clustering on the wound region to quantify the proportions of seven major colors present, giving clinicians an automated “tissue composition” report (for example, an infected ulcer might show higher percentages of yellow-green, representing exudate). Texture metrics (smooth vs. rough areas) can likewise hint at biofilm or slough. By correlating these color/texture features with infection, ML models can detect infections that manifest as changes in wound appearance. One limitation is that some infection signs like warmth or odor cannot be captured visually; however, visual proxies (e.g., increased redness, swelling) are used as surrogates. There is active research into multimodal imaging as well–for instance, combining standard photography with infrared thermography to highlight hotspots of inflammation. In summary, tissue and color analysis tools use computer vision to objectively identify purulent material, necrotic patches, and inflammatory coloration. These augmentations enhance the clinician’s ability to assess how advanced an infection is, beyond a simple yes/no classification.

### 4.3 Tools for monitoring temporal changes

Machine learning is also powering smart monitoring tools that track diabetic foot ulcers over time (van Netten et al., 2017). Because infections can develop or worsen in a matter of days, continuous monitoring is crucial–especially for patients at home or in rural areas. To this end, researchers and companies have developed smartphone-based applications that use ML to analyze serial wound images. These apps enable patients or caregivers to regularly photograph their ulcers, while built-in algorithms assess healing progression and warning signs of infection. For example, DFUCare is a mobile deep-learning platform that not only detects and classifies ulcers in images, but also measures wound size and analyzes color changes on successive visits. In a pilot study, DFUCare’s infection classifier achieved ∼80% accuracy and its ischemia detector ∼95% accuracy, while successfully measuring wound area within a few millimeters of error. By comparing each new image to prior ones, the system can flag if an ulcer is enlarging or if redness is increasing–potential harbingers of infection or deterioration.

Another example is the use of the Swift Skin and Wound app (Health Canada/FDA cleared) in remote patient care (Kong et al., 2021). This app uses computer-vision to calibrate wound measurements and allows patients to upload images to their provider. A recent case report demonstrated its utility: a high-risk patient with diabetic foot ulcers used the app to send images weekly, enabling clinicians to remotely monitor three episodes of infection and adjust treatment promptly (Kong et al., 2021). The app’s ML features provided objective wound size and appearance data, giving the clinician confidence in the healing trajectory and allowing intervention (e.g., starting antibiotics) at the earliest sign of recurrence. Overall, these monitoring tools leverage ML for trend analysis–they detect changes over time that might indicate an infection is not responding to treatment or that a dormant wound has become acutely infected. In resource-limited or rural settings, such smartphone apps can serve as an “early warning system,” alerting specialists to intervene before minor infections become limb-threatening. By increasing the frequency and quality of ulcer assessments outside the clinic, ML-powered monitoring reduces the burden on patients (fewer travel visits) while maintaining vigilance.

## 5 Clinical utility of ML tools in DFU care

**FIGURE 1:**
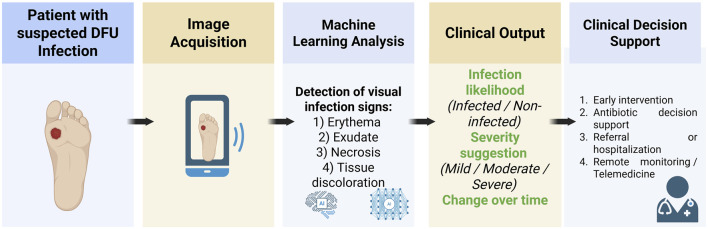
Conceptual workflow illustrating machine learning–assisted infection assessment in diabetic foot ulcers. Digital images of diabetic foot ulcers are acquired using standardized wound photography. Machine learning–based image analysis identifies visual features associated with infection, including erythema, exudate, necrosis, and tissue discoloration. The resulting output provides clinicians with decision-support information, facilitating early diagnosis, remote monitoring, and timely clinical intervention.

Machine learning tools for DFU infection assessment are increasingly finding practical roles in clinical care. Decision support is one clear use case: AI algorithms can assist clinicians by providing a second opinion on whether an ulcer is infected and how severe it might be. For instance, an ML system could automatically analyze a wound photo during a clinic visit and highlight regions of concern (e.g., spreading redness) to the clinician. This aids in triaging patients–ensuring those with signs of serious infection get prompt attention or referral to specialty care. In one 2024 report, an AI scoring system’s infection assessment closely matched senior dermatologist opinions and even outperformed junior doctors (Wang et al., 2024). Such a tool could be used in multidisciplinary foot clinics to improve consistency in diagnosing infection, thereby standardizing care. Additionally, by quantifying infection extent, ML decision-support systems may guide antibiotic stewardship (e.g., indicating if only mild local infection is present that could be managed with topical agents vs. a moderate-to-severe infection needing systemic antibiotics) (Figure 1).

Emerging studies also suggests that ML-driven interventions can positively impact clinical workflows and patient outcomes. By automating time-consuming aspects of wound assessment (measurement, documentation of tissue types, etc.), clinicians can focus more on treatment decisions and patient education. For example, the DFUCare platform allows a clinician to get a full analysis of an ulcer image (size, infection status, tissue breakdown) within seconds on a tablet or phone (Sendilraj et al., 2024). This not only saves the clinician’s time but also ensures that no aspect of the wound exam is overlooked. In telemedicine scenarios, these tools enable effective remote management: one case study reported that using an AI-enabled wound app for remote monitoring led to fewer in-person visits while still achieving healing, as the care team could intervene via telehealth at early signs of deterioration. Such remote-monitoring ML systems are especially useful for patients who have mobility issues or live far from specialty clinics. They effectively extend the clinical oversight beyond the hospital walls, which has been linked to reduced rates of severe complications like amputations in some early deployments. Moreover, ML tools can prioritize cases: in a home telemedicine program, images flagged by the algorithm as showing possible infection can be triaged for faster review, ensuring urgent cases are seen sooner.

## 6 Limitations and barriers to clinical adoption

Despite their considerable promise, current machine learning approaches for assessing diabetic foot ulcer (DFU) infection face several notable constraints that impede their transition into routine clinical use. One major challenge concerns dataset bias and generalizability. Many existing models have been trained on relatively narrow image repositories that do not reflect the diversity of real-world patients. Many commonly used datasets, including DFUC 2020, consist predominantly of images from patients with lighter skin tones. Consequently, algorithms trained on these datasets may demonstrate reduced sensitivity in individuals with darker skin pigmentation, where erythema and inflammatory colour changes are less visually apparent, increasing the risk of systematic infection under-detection in underrepresented populations. Furthermore, models built within a single institution or geographic region may fail to accommodate differences in clinical practice, camera equipment, or wound care standards elsewhere. To counter these shortcomings, researchers are exploring strategies such as multi-center dataset aggregation, data augmentation, and domain adaptation to broaden model applicability. Emerging methodologies like federated learning, which enables distributed model training without centralizing patient data, are also being investigated as scalable solutions to improve generalizability across varied populations.

Beyond dataset representativeness, image variability and data quality pose significant obstacles. Real-world wound photographs are often captured under inconsistent conditions, with imperfect lighting, variable focus, and visual obstructions such as dressings or blood. Machine learning systems are sensitive to such inconsistencies, and seemingly minor issues can distort model interpretation. Such environmental variability directly influences infection classification performance and may lead to false-positive or false-negative predictions, for example, when surface moisture is misinterpreted as purulent exudate or when suboptimal lighting obscures periwound erythema. For example, an ischemic ulcer may be misread as infected if lighting causes the tissue to appear abnormally pale, or moisture artefacts may falsely suggest purulence. Variability in how patients or clinicians position the camera introduces further noise, while imperfect annotation of ground truth infection boundaries compounds the problem. Infection labels used during training are frequently based on subjective clinical judgment or microbiological tests, each of which has limitations. Even experienced annotators may inconsistently delineate where infected tissue ends and healthy tissue begins—a known source of error in segmentation tasks. These challenges underscore the importance of standardized image capture protocols and high-quality labeling, yet adherence to such standards remains uneven across current applications.

Regulatory hurdles and workflow integration represent additional barriers. Translating an ML model from experimental validation to clinical deployment requires rigorous scrutiny by regulatory bodies such as the FDA or EMA. At present, most machine learning systems for diabetic foot infection detection remain investigational and have not obtained formal approval as diagnostic medical devices, reflecting the limited availability of large-scale prospective validation data and standardized imaging protocols. These agencies demand comprehensive evidence of safety, accuracy, and clinical effectiveness—requirements that few DFU infection AI tools have met to date. Although some related measurement applications have obtained device approval, full regulatory endorsement for infection diagnosis remains rare. Even when regulatory clearance is achieved, embedding AI tools into existing clinical workflows introduces logistical challenges. Clinics may need new equipment, software interfaces, or staff training, and busy practitioners may resist systems that require additional steps or increase cognitive load. Concerns about liability also linger, particularly when an AI’s recommendation conflicts with a clinician’s judgment or fails to detect an evolving infection. Seamless integration into electronic health record systems and user-centered interface design are essential to mitigate these issues and ensure that ML output supports rather than disrupts clinical decision-making.

A final barrier relates to clinician trust and the explainability of AI models. Many healthcare providers remain wary of systems whose internal reasoning they cannot interrogate. The «black box» nature of many deep learning architectures represents a substantial barrier to clinical endorsement, particularly when algorithmic outputs inform high-stakes decisions such as antibiotic initiation, surgical intervention, or amputation. When an algorithm labels a wound as infected without providing a coherent rationale, clinicians may discount or ignore the recommendation—especially in ambiguous cases where diagnostic uncertainty is already high. The opaque nature of deep learning exacerbates concerns about hidden biases and undetected errors, which pose substantial risks in a clinical context. To address these reservations, researchers are prioritizing the development of explainable AI. Tools such as Grad-CAM heatmaps visually highlight image regions that influenced the model’s prediction, enabling clinicians to assess whether the model’s focus aligns with their own observations. More sophisticated architectures, including explainability-centered models like XAI-FusionNet, aim to blend multi-scale features with interpretable outputs to generate both a prediction and its justification. Although these innovations represent meaningful progress, their real-world effectiveness in improving clinician confidence remains under evaluation. Ultimately, broad acceptance of ML-driven infection assessment will depend on demonstrating accuracy, transparency, fairness, and alignment with clinical reasoning. Cultivating trust through ongoing validation studies, education, and careful deployment strategies will be essential for transforming these tools from experimental prototypes into routine collaborators in patient care. Collectively, dataset bias, environmental image variability, regulatory constraints, and limited model explainability remain central challenges that must be addressed before machine learning–based infection assessment tools can be safely and equitably integrated into routine diabetic foot care.

## 7 Future perspectives

Looking ahead, the integration of machine learning into the care of diabetic foot ulcer (DFU) infections is expected to accelerate, driven by rapid technological innovation and evolving clinical demands. One important direction is the development of multimodal models. Instead of relying solely on wound images, future systems will incorporate diverse patient data to improve diagnostic and prognostic accuracy. A single photograph offers only a partial view of infection risk, whereas combining it with clinical variables such as body temperature, inflammatory markers, glycemic control, and past infection history could enable far more precise assessments. These models may also draw from sensor-based datasets, including thermal imaging of the foot or perfusion scans, allowing algorithms to differentiate between infectious cellulitis and benign inflammation. Early multimodal research in DFU outcome prediction supports this trajectory, demonstrating that clinical information combined with wound features enhances performance. Extending this approach to infection care opens the possibility of advanced decision-support systems that merge laboratory results or point-of-care diagnostics, like bacterial fluorescence imaging, with visual analysis to produce comprehensive infection risk scores. Achieving this vision will require large, carefully curated multimodal datasets.

Another major area of progress will revolve around explainable and human-centered artificial intelligence. Machine learning tools must not only be accurate but also trustworthy and intelligible to clinicians. Current efforts are shifting away from opaque, black-box architectures toward systems capable of justifying their predictions. These may include visual cues on wound images showing the features that influenced infection probability, narrative explanations such as noting measurable increases in redness over time, or case-based comparisons with similar wounds in the training set. These elements can align AI outputs with the cognitive processes clinicians already use in decision-making. Equally critical will be design approaches tailored to real clinical workflows. Future systems may adapt dynamically to clinician corrections, reflect local patient population characteristics, and evolve alongside practice patterns. The long-term aspiration is an AI assistant that enhances professional expertise, offers transparent rationale for its recommendations, and continually refines itself based on user feedback.

The widespread adoption of machine learning in DFU care will ultimately depend on rigorous validation. Large-scale, prospective, multi-center clinical trials will be required to determine whether AI-assisted infection assessment improves outcomes compared with standard care. These studies will investigate whether earlier interventions, reduced amputation rates, improved antibiotic stewardship, or faster healing can be achieved when clinicians use such tools. Real-world implementation across varied healthcare environments—from specialty wound clinics to primary care facilities and home-based monitoring programs—will test the robustness and generalizability of these systems. Economic evaluations are also expected as stakeholders seek evidence that AI solutions can reduce costs by preventing complications and hospital admissions. Over the next decade, expanding registries and trial data will help identify which machine learning approaches deliver meaningful clinical value and define how they should be integrated into care pathways.

In parallel, a new generation of smart wound care platforms is emerging, potentially transforming infection management through continuous monitoring and automated analysis. These platforms may unite sensing devices, software applications, and AI algorithms into a single ecosystem. Patients could use smart dressings equipped with embedded sensors capable of detecting biochemical changes associated with infection; the data would be transmitted to a smartphone application that analyzes both sensor readings and periodic photographs to flag early warning signs. Several elements of this scenario already exist in early forms, including telemedicine systems for automated wound photography and cloud-based analytical services, as well as experimental wound dressings able to signal bacterial activity. As innovation progresses, integration across multiple data streams—visual, thermal, biochemical—will be essential to generating a dynamic, real-time portrait of wound health. These platforms may link directly with electronic health records, enabling clinicians to incorporate AI-generated insights during consultations. Simplified versions, relying only on smartphone imaging and lightweight AI models, could profoundly expand access in resource-limited settings. Collectively, these advancements suggest the emergence of a digitally enabled wound care paradigm in which constant monitoring, intelligent algorithms, and clinician oversight converge to mitigate the burden of DFU infections and improve patient outcomes.

## 8 Conclusion

Machine learning is set to play a transformative role in the assessment of infections in diabetic foot ulcers. As reviewed, ML techniques can overcome many limitations of traditional visual inspection by providing objective, consistent, and rapid analysis of wound images. From classification models that detect infection with high sensitivity, to segmentation algorithms that quantify the extent of infection, to mobile apps enabling daily monitoring–these tools offer clinical decision support that can lead to earlier interventions and personalized care. Evidence to date suggests that when properly implemented, AI systems can match expert-level diagnostic accuracy for DFU infections. Perhaps most importantly, ML has the potential to extend specialist expertise to the point-of-care for any patient, thereby closing gaps in access and reducing delays in treating infected foot ulcers.

That said, significant gaps and challenges remain on the path to full clinical adoption. Many ML models have been developed and validated only in retrospective or experimental settings, and their real-world impact on patient outcomes is still unproven. Key issues like algorithm transparency, integration into clinical workflows, and ensuring generalizability across diverse patient populations must be addressed. Collaboration between clinicians, data scientists, and regulatory bodies will be crucial to refine these technologies and build trust in their use. An implementation roadmap for the coming years includes conducting robust clinical trials, obtaining regulatory approvals, training end-users, and establishing guidelines for when and how to use ML recommendations in DFU care.

In conclusion, machine learning-driven infection assessment for diabetic foot ulcers offers a promising solution to longstanding clinical challenges of subjectivity and delayed diagnosis. By augmenting clinical decision-making with data-driven insights, ML can help identify infections earlier, guide appropriate therapies (like antibiotics or surgical referral), and ultimately improve limb salvage rates. The field is moving rapidly–what was a novel research idea a few years ago is now nearing translation into practice. With continued interdisciplinary effort, ML tools are poised to become an integral part of diabetic foot ulcer management, ushering in a new era of precision wound care where preventable amputations due to unrecognized infections become increasingly rare. The remaining journey involves scaling up validation and carefully integrating these innovations into the healthcare ecosystem, but the potential benefits for patients with diabetes are immense. Each step taken to refine and adopt these technologies is a step toward better outcomes and quality of life for those at risk of diabetic foot complications.

## References

[B1] Almufadi N. Alhasson H. F. (2024). Classification of diabetic foot ulcers from images using machine learning approach. Diagn. (Basel, Switz.) 14 (16), 1807. 10.3390/diagnostics14161807 39202295 PMC11353632

[B2] Álvaro-Afonso F. J. Lázaro-Martínez J. L. Aragón-Sánchez J. García-Morales E. Cecilia-Matilla A. Beneit-Montesinos J. V. (2013). Interobserver and intraobserver reproducibility of plain X-rays in the diagnosis of diabetic foot osteomyelitis. International Journal Lower Extremity Wounds 12 (1), 12–15. 10.1177/1534734612474304 23378514

[B3] Álvaro-Afonso F. J. Tardáguila-García A. López-Moral M. Sanz-Corbalán I. García-Morales E. Lázaro-Martínez J. L. (2025). Using artificial intelligence for detecting diabetic foot osteomyelitis: validation of deep learning model for plain radiograph. Interpretation 15 (15), 8583. 10.3390/app15158583

[B4] Armstrong D. G. Swerdlow M. A. Armstrong A. A. Conte M. S. Padula W. V. Bus S. A. (2020). Five year mortality and direct costs of care for people with diabetic foot complications are comparable to cancer. J. Foot Ankle Research 13 (1), 16. 10.1186/s13047-020-00383-2 32209136 PMC7092527

[B5] Armstrong D. G. Tan T. W. Boulton A. J. M. Bus S. A. (2023). Diabetic foot ulcers: a review. Jama 330 (1), 62–75. 10.1001/jama.2023.10578 37395769 PMC10723802

[B6] Basiri R. (2025). “Applications of generative AI in diabetic foot ulcer treatment,” in Diabetic foot - advanced methods of management. 10.5772/intechopen.1012472

[B7] Bowling F. L. King L. Paterson J. A. Hu J. Lipsky B. A. Matthews D. R. (2011). Remote assessment of diabetic foot ulcers using a novel wound imaging system. Wound Repair Regeneration Official Publication Wound Heal. Soc. Eur. Tissue Repair Soc. 19 (1), 25–30. 10.1111/j.1524-475X.2010.00645.x 21134035

[B8] Brown S. T. Collier H. Askew L. Gilberts R. M. Sharples L. D. Nixon J. (2025). Diabetic foot ulcer photography study: a study within a trial to assess the reliability of two-dimensional (2D) photography for the assessment of ulcer healing in patients with diabetes-related foot ulcers-protocol paper. BMJ Open 15 (1), e090299. 10.1136/bmjopen-2024-090299 39788763 PMC11752009

[B9] Cassidy B. Kendrick C. Reeves N. Pappachan J. O’Shea C. Armstrong D. (2022). Diabetic foot ulcer grand challenge 2021: evaluation and summary. 90–105.

[B10] Cazzell S. Moyer P. M. Samsell B. Dorsch K. McLean J. Moore M. A. (2019). A prospective, multicenter, single-arm clinical trial for treatment of complex diabetic foot ulcers with deep exposure using acellular dermal matrix. Adv. Skin and Wound Care 32 (9), 409–415. 10.1097/01.ASW.0000569132.38449.c0 31361269 PMC7328871

[B11] Chuan F. Tang K. Jiang P. Zhou B. He X. (2015). Reliability and validity of the perfusion, extent, depth, infection and sensation (PEDIS) classification system and score in patients with diabetic foot ulcer. PLoS One 10 (4), e0124739. 10.1371/journal.pone.0124739 25875097 PMC4395335

[B12] Craus S. Mula A. Coppini D. V. (2023). The foot in diabetes - a reminder of an ever-present risk. Clin. Medicine Lond. Engl. 23 (3), 228–233. 10.7861/clinmed2022-0489 37197806 PMC11046562

[B13] Debnath S. Khurana A. Senbagavalli M. Naik S. Chandra Patni J. Mishra P. K. (2025). Sustainable AI for diabetic foot ulcer detection: a deep learning approach for early diagnosis. Discov. Appl. Sci. 7 (9), 1012. 10.1007/s42452-025-07601-1

[B14] Dias de Oliveira T. C. de Oliveira A. F. Araújo L. C. Moreira de Sena M. P. Fagundes V. C. Rabelo P. P. A. (2025). Digital health technologies for diabetic foot ulcers: a systematic review of clinical evidence, access inequities, and. Public Health Integr. 22 (9), 1430. 10.3390/ijerph22091430 41007574 PMC12469766

[B15] Edmonds M. Manu C. Vas P. (2021). The current burden of diabetic foot disease. J. Clinical Orthopaedics Trauma 17, 88–93. 10.1016/j.jcot.2021.01.017 33680841 PMC7919962

[B16] Elawady K. (2025). Comprehensive overview of foot and ankle trauma - diagnosis, treatment, sequels and rehabilitation.

[B17] Foltynski P. Ladyzynski P. Migalska-Musial K. Sabalinska S. Ciechanowska A. Wojcicki J. (2011). A new imaging and data transmitting device for telemonitoring of diabetic foot syndrome patients. Diabetes Technol. Ther. 13 (8), 861–867. 10.1089/dia.2011.0004 21568750

[B18] Foltynski P. Kruszewska K. Krakowiecki A. Czarkowska-Paczek B. Ladyzynski P. (2025). Artificial intelligence models for wound infection recognition and their comparison with human results. Biocybern. Biomed. Eng. 45 (3), 572–579. 10.1016/j.bbe.2025.08.003

[B19] Gherardi A. Bo W. Demirbas A. Zhan Y. Xu W. (2025). Hyperspectral reconstruction for mobile diabetic foot blood perfusion monitoring. BMC Artificial Intelligence 1 (1), 10. 10.1186/s44398-025-00011-8 40994833 PMC12454483

[B20] Goyal M. Reeves N. D. Rajbhandari S. Ahmad N. Wang C. Yap M. H. (2020). Recognition of ischaemia and infection in diabetic foot ulcers: dataset and techniques. Comput. Biol. Med. 117, 103616. 10.1016/j.compbiomed.2020.103616 32072964

[B21] Guan H. Wang Y. Niu P. Zhang Y. Zhang Y. Miao R. (2024). The role of machine learning in advancing diabetic foot: a review. Front. Endocrinol. 15, 2024. 10.3389/fendo.2024.1325434 38742201 PMC11089132

[B22] Harrison J. (2023). A scoping review exploring the confidence of healthcare professionals in assessing all skin tones. Br. Paramed. J. 8 (2), 18–28. 10.29045/14784726.2023.9.8.2.18 37674918 PMC10477824

[B23] Hazenberg C. E. van Baal J. G. Manning E. Bril A. Bus S. A. (2010). The validity and reliability of diagnosing foot ulcers and pre-ulcerative lesions in diabetes using advanced digital photography. Diabetes Technol. Ther. 12 (12), 1011–1017. 10.1089/dia.2010.0088 21128848

[B24] Karthik R. Ajay A. Jhalani A. Ballari K. Suganthi K. (2025). An explainable deep learning model for diabetic foot ulcer classification using swin transformer and efficient multi-scale attention-driven network. Sci. Rep. 15 (1), 4057. 10.1038/s41598-025-87519-1 39900977 PMC11791195

[B25] Kong L. Y. Ramirez-GarciaLuna J. L. Fraser R. D. J. Wang S. C. (2021). A 57-Year-Old man with type 1 diabetes mellitus and a chronic foot ulcer successfully managed with a remote patient-facing wound care smartphone application. Am. Journal Case Reports 22, e933879. 10.12659/AJCR.933879 34910717 PMC8689370

[B26] Lipsky B. A. Berendt A. R. Deery H. G. Embil J. M. Joseph W. S. Karchmer A. W. (2004). Diagnosis and treatment of diabetic foot infections. Clin. Infectious Diseases An Official Publication Infect. Dis. Soc. Am. 39 (7), 885–910. 10.1086/424846 15472838

[B27] Lipsky B. A. Berendt A. R. Cornia P. B. Pile J. C. Peters E. J. Armstrong D. G. (2012). Infectious diseases society of America clinical practice guideline for the diagnosis and treatment of diabetic foot infections. Clin. Infectious Diseases An Official Publication Infect. Dis. Soc. Am. 54 (12), e132–e173. 10.1093/cid/cis346 22619242

[B28] Liu Z. John J. Agu E. (2022). Diabetic foot ulcer ischemia and infection classification using EfficientNet deep learning models. IEEE Open J. Eng. Med. Biol. 3, 189–201. 10.1109/OJEMB.2022.3219725 36660100 PMC9842228

[B29] Llewellyn A. Kraft J. Holton C. Harden M. Simmonds M. (2020). Imaging for detection of osteomyelitis in people with diabetic foot ulcers: a systematic review and meta-analysis. Eur. Journal Radiology 131, 109215. 10.1016/j.ejrad.2020.109215 32862106

[B30] Luu I. Y. Hong A. T. Lin F. Shin L. Shih C.-D. Han S. M. (2025). Trends of lower-limb complications in patients with type 2 diabetes mellitus during the COVID-19 pandemic. Diabetes Res. Clin. Pract. 226, 112331. 10.1016/j.diabres.2025.112331 40543573 PMC12263163

[B31] Méndez N. G. Aguilera M. F. G. Muñoz E. Á. Rivadeneira J. Cabrera P. J. B. Totomoch-Serra A. (2025). New technologies applied in self-care to patients with diabetic foot ulcers: a scoping review. Diabetol. Metab. Syndr. 17 (1), 262. 10.1186/s13098-025-01822-5 40646659 PMC12255125

[B32] Murphy R. X. Jr. Bain M. A. Wasser T. E. Wilson E. Okunski W. J. (2006). The reliability of digital imaging in the remote assessment of wounds: defining a standard. Ann. Plastic Surgery 56 (4), 431–436. 10.1097/01.sap.0000202146.92893.6a 16557079

[B33] National Institute for Health and Care Excellence: Guidelines (2023). Diabetic foot problems: prevention and management. London: National Institute for Health and Care Excellence (NICE) Copyright © NICE. 32045177

[B34] Onuh O. C. Brydges H. T. Nasr H. Savage E. Gorenstein S. Chiu E. (2022). Capturing essentials in wound photography past, present, and future: a proposed algorithm for standardization. Adv. Skin and Wound Care 35 (9), 483–492. 10.1097/01.ASW.0000852564.21370.a4 35993857

[B35] Ramirez-Acuña J. Cardenas Cadena S. Marquez-Salas P. Garza-Veloz I. Perez-Favila A. Cid-Báez M. A. (2019). Diabetic foot ulcers: current advances in antimicrobial therapies and emerging treatments. Antibiotics 8, 193. 10.3390/antibiotics8040193 31652990 PMC6963879

[B36] Rasmussen B. S. Froekjaer J. Joergensen L. B. Halekoh U. Yderstraede K. B. (2015). Validation of a new imaging device for telemedical ulcer monitoring. Skin Research Technology Official Journal Int. Soc. Bioeng. Skin (ISBS) Int. Soc. Digital Imaging Skin (ISDIS) Int. Soc. Skin Imaging (ISSI) 21 (4), 485–492. 10.1111/srt.12218 25801649

[B37] Rathore P. S. Kumar A. Nandal A. Dhaka A. Sharma A. K. (2025). A feature explainability-based deep learning technique for diabetic foot ulcer identification. Sci. Rep. 15 (1), 6758. 10.1038/s41598-025-90780-z 40000748 PMC11862115

[B38] Sait A. R. W. Nagaraj R. (2025). Diabetic foot ulcers detection model using a hybrid convolutional neural networks–vision transformers. Diagn. (Basel). 15 (6), 736. 10.3390/diagnostics15060736 40150079 PMC11941693

[B39] Sari N. N. Ngo Q. C. Pah N. D. Ogrin R. Ekinci E. Hourani A. (2025). Non-invasive imaging techniques for predicting healing status of diabetic foot ulcers: a ten-year systematic review. Front. Med. Technol. 7, 1648973. 10.3389/fmedt.2025.1648973 41089884 PMC12515947

[B40] Schaper N. C. van Netten J. J. Apelqvist J. Bus S. A. Hinchliffe R. J. Lipsky B. A. (2020). Practical guidelines on the prevention and management of diabetic foot disease (IWGDF 2019 update). Diabetes/metabolism Research Reviews 36 (Suppl. 1), e3266. 10.1002/dmrr.3266 32176447

[B41] Schaper N. C. van Netten J. J. Apelqvist J. Bus S. A. Fitridge R. Game F. (2024). Practical guidelines on the prevention and management of diabetes-related foot disease (IWGDF 2023 update). Diabetes/Metabolism Research Reviews 40 (3), e3657. 10.1002/dmrr.3657 37243927

[B42] Sen P. Demirdal T. (2020). Evaluation of mortality risk factors in diabetic foot infections. Int. Wound Journal 17 (4), 880–889. 10.1111/iwj.13343 32196927 PMC7949473

[B43] Sendilraj V. Pilcher W. Choi D. Bhasin A. Bhadada A. Bhadadaa S. K. (2024). DFUCare: deep learning platform for diabetic foot ulcer detection, analysis, and monitoring. Front. Endocrinol. 15, 2024. 10.3389/fendo.2024.1386613 39381435 PMC11460545

[B44] Senneville É. Albalawi Z. van Asten S. A. Abbas Z. G. Allison G. Aragón-Sánchez J. (2024). IWGDF/IDSA guidelines on the diagnosis and treatment of diabetes-related foot infections (IWGDF/IDSA 2023). Diabetes/metabolism Research Reviews 40 (3), e3687. 10.1002/dmrr.3687 37779323

[B45] Shen D. Wu G. Suk H. I. (2017). Deep learning in medical image analysis. Annu. Rev. Biomed. Eng. 19, 221–248. 10.1146/annurev-bioeng-071516-044442 28301734 PMC5479722

[B46] Søndergaard S. F. Vestergaard E. G. Andersen A. B. Kolbaek R. Dahl M. Høgh A. (2023). How patients with diabetic foot ulcers experience telemedicine solutions: a scoping review. Int. Wound J. 20 (5), 1796–1810. 10.1111/iwj.14026 36453130 PMC10088844

[B47] Spinazzola E. Picaud G. Becchi S. Pittarello M. Ricci E. Chaumont M. (2025). Chronic ulcers healing prediction through machine learning approaches: preliminary results on diabetic foot ulcers case. Study 14 (9), 2943. 10.3390/jcm14092943 40363975 PMC12072452

[B48] Totty J. P. Harwood A. E. Wallace T. Smith G. E. Chetter I. C. (2018). Use of photograph-based telemedicine in postoperative wound assessment to diagnose or exclude surgical site infection. J. Wound Care 27 (3), 128–135. 10.12968/jowc.2018.27.3.128 29509108

[B49] Tulloch J. Zamani R. Akrami MJIA (2020). Machine learning in the prevention, diagnosis and management of diabetic foot ulcers. A Systematic Review 8, 198977–199000. 10.1109/access.2020.3035327

[B50] van Netten J. J. Clark D. Lazzarini P. A. Janda M. Reed L. F. (2017). The validity and reliability of remote diabetic foot ulcer assessment using mobile phone images. Sci. Rep. 7 (1), 9480. 10.1038/s41598-017-09828-4 28842686 PMC5573347

[B51] van Netten J. J. Bus S. A. Apelqvist J. Chen P. Chuter V. Fitridge R. (2024). Definitions and criteria for diabetes-related foot disease (IWGDF 2023 update). Diabetes. Metab. Res. Rev. 40 (3), e3654. 10.1002/dmrr.3654 37186781

[B52] Wang L. Pedersen P. C. Strong D. M. Tulu B. Agu E. Ignotz R. (2015). Smartphone-based wound assessment system for patients with diabetes. IEEE Trans. Biomed. Eng. 62 (2), 477–488. 10.1109/TBME.2014.2358632 25248175

[B53] Wang Z. Tan X. Xue Y. Xiao C. Yue K. Lin K. (2024). Smart diabetic foot ulcer scoring system. Sci. Rep. 14 (1), 11588. 10.1038/s41598-024-62076-1 38773207 PMC11109117

[B54] Weigelt M. A. Lev-Tov H. A. Tomic-Canic M. Lee W. D. Williams R. Strasfeld D. (2022). Advanced wound diagnostics: toward transforming wound care into precision medicine. Adv. Wound Care 11 (6), 330–359. 10.1089/wound.2020.1319 34128387 PMC8982127

[B55] Wukich D. K. Hobizal K. B. Brooks M. M. (2013). Severity of diabetic foot infection and rate of limb salvage. Foot and Ankle International 34 (3), 351–358. 10.1177/1071100712467980 23520292 PMC4016951

[B56] Xu Y. Han K. Zhou Y. Wu J. Xie X. Xiang W. (2022). Classification of diabetic foot ulcers using class knowledge banks. Front. Bioeng. Biotechnol. 9, 2021. 10.3389/fbioe.2021.811028 35295708 PMC8918844

[B57] Yap M. H. Ng C.-C. Chatwin K. Abbott C. Bowling F. Boulton A. (2015). Computer vision algorithms in the detection of diabetic foot ulceration: a new paradigm for diabetic foot care? J. Diabetes Sci. Technol. 10, 612–613. 10.1177/1932296815611425 26468134 PMC4773968

[B58] Yap M. H. Hachiuma R. Alavi A. Brüngel R. Cassidy B. Goyal M. (2021). Deep learning in diabetic foot ulcers detection: a comprehensive evaluation. Comput. Biol. Med. 135, 104596. 10.1016/j.compbiomed.2021.104596 34247133

[B59] Yasin P. Dong S. Aizezi Z. Yimit Y. Yusufu A. Yakufu M. (2025). Explainable machine learning for differential diagnosis of diabetic foot infection and osteomyelitis: a two-center study and clinically applicable web calculator using routine blood biomarkers. BMC Med. Inf. Decis. Mak. 25 (1), 420. 10.1186/s12911-025-03236-9 41219967 PMC12606877

[B60] Yogapriya J. Chandran V. Sumithra M. G. Elakkiya B. Shamila Ebenezer A. Suresh Gnana Dhas C. (2022). Automated detection of infection in diabetic foot ulcer images using convolutional neural network. J. Healthc. Eng. 2022, 2349849. 10.1155/2022/2349849 35432819 PMC9007637

[B61] Zhou G.-X. Tao Y.-K. Hou J.-Z. Zhu H.-J. Xiao L. Zhao N. (2025). Construction and validation of a deep learning-based diagnostic model for segmentation and classification of diabetic foot. Front. Endocrinol. 16, 2025. 10.3389/fendo.2025.1543192 40270716 PMC12014428

[B62] Zhu G. Fu Z. Guo G. (2025). Applications and prospects of artificial intelligence in wound healing. Regenes. Repair Rehabil. 1 (4), 12–19. 10.1016/j.rerere.2025.08.001

